# Epidemiology and treatment of forearm fractures in Swedish children and adolescents

**DOI:** 10.1007/s00068-025-02986-5

**Published:** 2025-11-24

**Authors:** Yasmin D. Hailer, Moa Elvefors Wallin, Olof Wolf

**Affiliations:** Department of Surgical Sciences, Section of Pediatric Orthopedic Surgery, Uppsala University, Uppsala, Sweden

**Keywords:** Forearm fractures, Demography, Swedish Fracture Register, Injury mechanism, Seasonal variation, Treatment

## Abstract

**Background:**

Previous studies, mainly single-center, on pediatric forearm fractures have shown that boys sustain fractures more frequently than girls. The most common injury mechanisms are falls and fractures occur more frequently during the summer. Most pediatric forearm fractures are treated non-surgically. This observational study aimed to present the demographics of pediatric forearm fractures in Sweden using the Swedish Fracture Register (SFR).

**Methods:**

We included pediatric patients < 16 years with a registered forearm fracture in the SFR between January 1st, 2015, and December 31st, 2019. We analyzed age, sex, injury mechanisms, anatomical location of the fracture, type of fracture, and initial treatment.

**Results:**

This study included 26,587 patients with 27,987 forearm fractures. Boys sustained a forearm fracture more frequently than girls. The boys were also significantly older. The mean age of all patients was 9.6 years. Distal fractures were most common, followed by diaphyseal and proximal fractures. Most of the fractures were torus fractures sustained to the distal radial metaphysis. Falls were the dominating injury mechanism. Most of the fractures occurred during playtime or sports activities. The most common injury place was registered as “unspecified”, followed by “sports facilities”. The highest fracture occurrence was observed during the summer. Most of the fractures were treated non-surgically.

**Conclusions:**

Forearm fractures are a common injury in children, with 75% affecting the distal forearm. The dominant injury mechanism was falls, regardless of age. Both sex and age affect the risks of sustaining a fracture, with boys and older children being at a greater risk. Our findings highlight the need for strengthened safety measures together with fall education.

## Introduction

 Approximately 10–25% of all injuries sustained by children are fractures [1, 2]. Forearm fractures account for 30–40% of all pediatric fractures. The most common fracture location is the distal forearm, representing 22–33% [3–5].

According to statistics from the National Board of Health and Welfare in Sweden [6], approximately 13,000 children per year between 0 and 14 years of age 2015–2022 sustain a forearm fracture, which is about 720/100,000 annually in this age group.

Most studies report a peak age for sustaining a forearm fracture of 10–11 years for girls and 12–13 years for boys [1]. Several studies agree that boys are more prone to sustaining a forearm fracture compared to girls [3, 7]. The most common injury mechanisms that cause forearm fractures are falls, traffic accidents, and during sports activities [7–9]. Some international studies have discovered an incidence peak of forearm fractures in the summer [10–12]. Most pediatric forearm fractures are treated non-surgically with closed reduction and/or casting [13–15]. Surgical methods used for internal fixation are pins, intramedullary nailing with elastic nails, and open reduction in combination with plates. Over the last 20 years, treatment with closed reduction in combination with intramedullary nailing or Kirschner-wires has increased, whereas open reduction in combination with plates has decreased [16].

Many published studies are either single-center studies or register studies based on a patient register, where diagnosis and treatment methods are less detailed, and do not include fracture classification. Further, many studies have either a focus on fractures in children in general or only fractures of one segment.

This study aimed to investigate the epidemiology and treatment of forearm fractures in children and answer the following research questions:

1. What is the demography for pediatric forearm fractures registered in the Swedish Fracture Register (SFR), concerning age, sex, fracture types, injury mechanisms, and injury activity?
2. Is there any seasonal variation in forearm fractures?
3. What are the most common primary treatment methods per fracture location and type?
4. Is there a changing trend in the treatment methods during the study period?

## Methods

### Study design and setting

#### Swedish Fracture Register (SFR)

This is an observational study from the SFR, a national quality register including fractures in adults since 2011 and in children since 2015. The treating physician registers the patient data (age and sex), injury mechanism, type of fracture (AO-classification), and treatment, both non-surgical and surgical [17, 18]. Only patients with Swedish personal identification numbers and fractures treated in Sweden are registered.

The fractures are classified using ICD-10 and the AO Pediatric Comprehensive Classification of Long-Bone Fractures (PCCF), which categorize the fractures based on the anatomical location, fracture pattern, and grade of severity.

### Study population

We extracted data on all pediatric patients < 16 years at injury with a forearm fracture (ICD S52) registered in the SFR with injury date between January 1 st, 2015, and December 31 st, 2019.

### Outcome variables

The outcome variables used were sex, age, anatomic location, fracture classification, injury mechanism (cause, activity, place), injury type, open/closed fracture, month of injury (season), and initial treatment.

The anatomic location of the fractures was divided into proximal, diaphyseal, or distal fractures, further into epiphyseal, metaphyseal, and diaphyseal. In addition, the PCCF-classification was used to categorize the fracture pattern.

Injury mechanisms were divided into falls, transport accidents, exposure to mechanical forces, exposure to living forces, and others, which included pathological-, stress-, and spontaneous fractures, assault, self-harm, and unspecified (UNS).

In the SFR, there are 9 categories for the place of injury: home, sports facility, school/hospital/public premises, unspecified, and others. The other category included street/road, store area/service area, industrial area/construction, agricultural area, other specified, and institutional housing.

Injury activity was divided into sports, play, unspecified, and others. The other category included acquisition work, rest/hygiene/meal, and other specified.

The injury type was divided into high-energy or low-energy trauma and was registered by the treating orthopedic surgeon. According to the SFR, low-energy trauma is considered to be same-level falls. Traffic accidents or falls from a height are considered as high-energy trauma. Sports injuries can be high or low energy trauma depending on the fracture pattern. Gustilo-Anderson (G-A) classification was used for open fractures.

Treatment was divided into two categories: non-surgical and surgical. The surgical method was then specified, and methods used were internal fixation with pins, intramedullary nails, fixation with screws, plates, bioimplant, other/combination methods or other surgical methods.

The seasonal variation was categorized according to the seasons: spring (Mar-May), summer (Jun-Aug), autumn (Sep-Nov), and winter (Dec-Feb).

### Statistics

Descriptive statistical methods such as means, percentages, and standard deviations were used to analyze continuous data. The normality of continuous data was ensured by the Shapiro-Wilk test and the test of skewness. The independent t-test was used to compare the means of continuous variables between the groups. The Chi-square test was used to compare groups with categorical variables. Multivariable logistic regression was used to estimate the odds ratio between the fractures and variables such as age, sex, treatment, and seasonal variation. The P-value for statistical significance was set to *p* < 0.05. All statistical analyses were performed using R Software package 4.4.2 (2024-10−31) [19].

## Results

### Population characteristics

26,587 patients were identified with 27,987 fractures, including 442 refractures. 14 patients died during the study period, unrelated to the fracture.

**Fig. 1:**
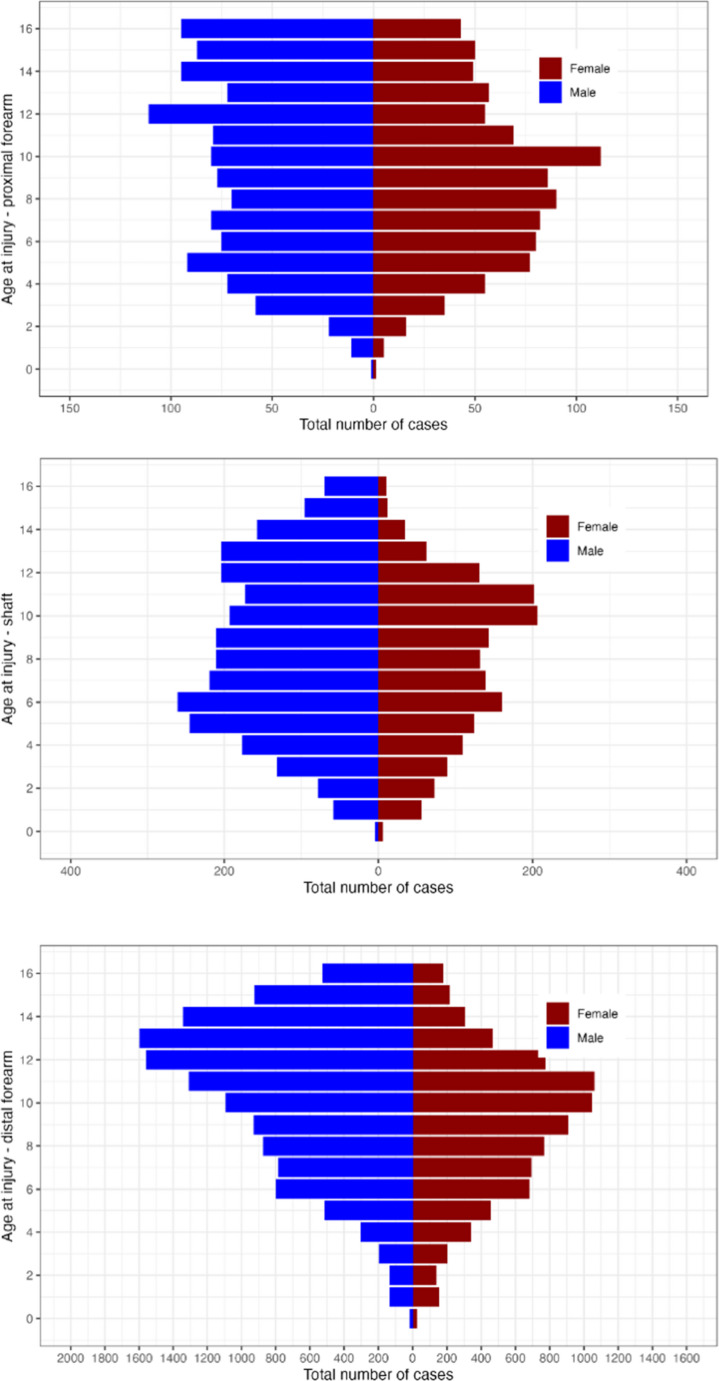
Forearm fracture (*n* = 27 987) occurrence in children < 16 years registered in the SFR 2015-2019 divided by sex and age for each anatomical segment (top: proximal forearm; middle: shaft; bottom: distal forearm)

Boys sustained 60.5% of the fractures, and they were statistically significantly older than girls, with a mean age of 10.1 (SD: 3.7) for boys and a mean age for girls 8.8 (SD: 3.4). Table 1 presents the characteristics of the patients of each fractured segment. Only distal fractures had a peak in boys at the ages of 12–13 years. Fractures in other segments were more evenly distributed (Fig. 1). In girls, the peak in all segments was around 10 years of age. The distal forearm was the most fractured segment, and among the distal forearm fractures, the distal radius was most common, with 71.3%.

**Table 1:** The demographics and treatment of each fractured segment of the forearm. SFR 2015–2019

	Proximal forearm (*n* = 2 139) 7.6%	Diaphyseal foream (*n* = 4 385) 15.7%	Distal foream (*n* = 21 463) 76.7%	Overall (*n* = 27 987) 100%
%				
Age				
Mean (SD)	9.36 (3.98)	8.26 (3.75)	9.89 (3.53)	9.59 (3.65)
**Sex**				
Male	1 177 (55.0%)	2 697 (61.5%)	13 045 (60.8%)	16 919 (60.5%)
Female	962 (45.0%)	1 688 (38.5%)	8 418 (39.2%)	11 068 (39.5%)

*Note: Surgical treatment includes closed reduction during general anesthesia

.

## Type of fracture

The most common fracture type for the whole forearm, classified by the PCCF-system, was the torus fracture, accounting for 44% of all fractures. This was also the case for the proximal and distal forearm fractures. The most common fracture type for diaphyseal fractures was complete, transverse, oblique, or spiral fractures with two or multi-fragment fractures. Table 2 presents the distribution of the five most common fracture types classified by the PCCF-system for each segment. 12% (*n* = 3378) were fractures engaging the physis, classified according to the Salter-Harris Classification [20].

**Table 2:** The distribution of the five most common fracture types in each forearm segment. SFR 2015–2019

Proximal fractures	Cases (*n* = 2 139)
Torus fracture	409 (19.1%)
Salter-Harris II	381 (17.8%)
Salter-Harris I	273 (12.8%)
Complete fracture, two fragment or more	213 (10.0%)
Undisplaced fracture	205 (9.6%)
Others	1494 (30.7)
**Diaphyseal fractures**	**Cases**(*n* = 4 385)
Complete, transverse, oblique or spiral fracture two or multi-fragment fracture	2 531 (57.7%)
Greenstick fracture	1 207 (27.5%)
Bowing of the bone	256 (5.8%)
Complete transverse or oblique radius fracture with bowing of the ulna	154 (3.5%)
Monteggia, ulna fracture with dislocation of the radial head	78 (1.8%)
Others	159 (3,7%)
**Distal fractures**	**Cases** (*n* = 21 463)
Torus fracture	11 848 (55.2%)
Complete fracture	5 414 (25.2%)
Salter-Harris II	1 636 (7.6%)
Torus fracture with avulsion of the styloid process	441 (2.1%)
Salter-Harris I	405 (1.9%)
Others	1719 (8.0%)

### Energy type and open fractures

78.3% of the fractures were due to a low-energy trauma. 0.9% (*n* = 247) of the fractures were open. Diaphyseal forearm fractures were the most common location, with 4.4% (192/4385) being open fractures. Gustilo-Anderson classification type 1 was most common (*n* = 183). Table 3 presents the injury and Gustilo-Andersson type together with the distribution for open fractures for each fractured segment.

**Table 3:** The distribution of injury type, open fractures, and Gustilo-Andersson classification per fractured segment of the forearm. SFR 2015–2019

	Proximal forearm (*n* = 2 139)	Diaphyseal foream (*n* = 4 385)	Distal foream (*n* = 21 463)	Overall (*n* = 27 987)
Injury type				
High energy trauma	131 (6.1%)	389 (8.9%)	1 197 (5.6%)	1 717 (6.1%)
Low energy trauma	1 624 (75.9%)	3 308 (75.4%)	16 976 (79.1%)	21 908 (78.3%)
Unknown/not applicable	142 (6.6%)	263 (5.8%)	1 209 (5.6%)	1 607 (5.7%)
Missing data	242 (11.3%)	432 (9.9%)	2 081 (9.7%)	2 755 (9.5%)
**Open fracture**				
Yes	4 (0.2%)	192 (4.4%)	51 (0.2%)	247 (0.9%)
No	2 135 (99.8%)	4 193 (95.6%)	21 412 (99.8%)	27 740 (99.1%)
**Gustilo-Anderson type**				
Type I	2 (0.1%)	152 (3.5%)	29 (0.1%)	183 (0.7%)
Type II	-	17 (0.4%)	*n* = 6	23 (0.1%)
Type IIIa	2 (0.1%)	7 (0.2%)	*n* = 8	17 (0.1%)
Type IIIb	-	*n* = 1	-	*n* = 1
Type IIIc	-	-	*n* = 1	*n* = 1
Unknown	-	15 (0.3%)	*n* = 7	22 (0.1%)

## Injury mechanism

### Cause

Across all ages, falls were the primary cause of fractures, comprising 74.4% of cases. The most common reasons for the children falling were stumbles, falls from play equipment, or falls during ice skating/snowboarding, etc. (Table 4). We identified 14 patients where the injury mechanism was assault. All children in this category were older than 7 years.

**Table 4:** The distribution of injury mechanisms categorized by cause, location, and activity for each forearm fracture segment. SFR 2015–2019

	Proximal forearm (*n* = 2 139)	Diaphyseal forearm (*n* = 4 385)	Distal forearm (*n* = 21 463)	Overall (*n* = 27 987)
Cause				
Fall	1 657 (77.5%)	3 572 (81.5%)	15 600 (72.7%)	20 829 (74.4%)
Transport accident	296 (13.8%)	402 (9.2%)	2 680 (12.5%)	3 378 (12.1%)
Mechanical forces	47 (2.2%)	139 (3.2%)	1 765 (8.2%)	1 951 (7.0%)
Living forces	59 (2.8%)	141 (3.2%)	759 (3.5%)	959 (3.4%)
Other	5 (0.2%)	24 (0.5%)	68 (0.3%)	97 (0.3%)
Missing data	75 (3.5%)	107 (2.4%)	591 (2.8%)	773 (2.8%)
**Place**				
Sports facilities	239 (11.2%)	636 (14.5%)	4 931 (23%)	5 806 (20.7%)
At home	536 (25.1%)	1 282 (29.2%)	3 505 (16.3%)	5 323 (19.0%)
School/hospital/public premises	314 (14.7%)	679 (15.5%)	3 042 (14.2%)	4 035 (14.4%)
Unspecified	547 (25.6%)	1 031 (23.5%)	5 666 (26.4%)	7 244 (25.9%)
Other	132 (6.1%)	248 (5.6%)	616 (4.8%)	1 428 (5.1%)
Missing data	371 (17.3%)	509 (11.6%)	3 271 (15.2%)	4 151 (14.8%)
**Activity**				
Playtime	922 (43.1%)	2 150 (49.0%)	7 495 (34.9%)	10 567 (37.8%)
Sports-related	313 (14.6%)	768 (17.5%)	5 591 (26.0%)	6 672 (23.8%)
Other	44 (2.0%)	89 (1.9%)	399 (1.8%)	532 (1.9%)
Unspecified	489 (22.9%)	869 (19.8%)	4 707 (21.9%)	6 065 (21.7%)
Missing data	371 (17.3%)	509 (11.6%)	3 271 (15.2%)	4 151 (14.8%)

### Activity and place

Most of the fractures were sustained during playtime (37.8%) or during sports (23.8%), (Table 4). Fractures sustained during playtime decreased with a higher age, while fractures related to sports activities increased with age.

In many cases, the place of injury was unspecified. When specified, the most common places where the fracture occurred were sports facilities and at home (Table 4). Fractures sustained at home and school/hospital/public premises increased with age, with a fracture occurrence peak at age 6 and then gradually decreased. The sports facilities were a common place to sustain a forearm fracture for older children, with a fracture occurrence peak at age 13.

### Seasonal variation

**Fig. 2:**
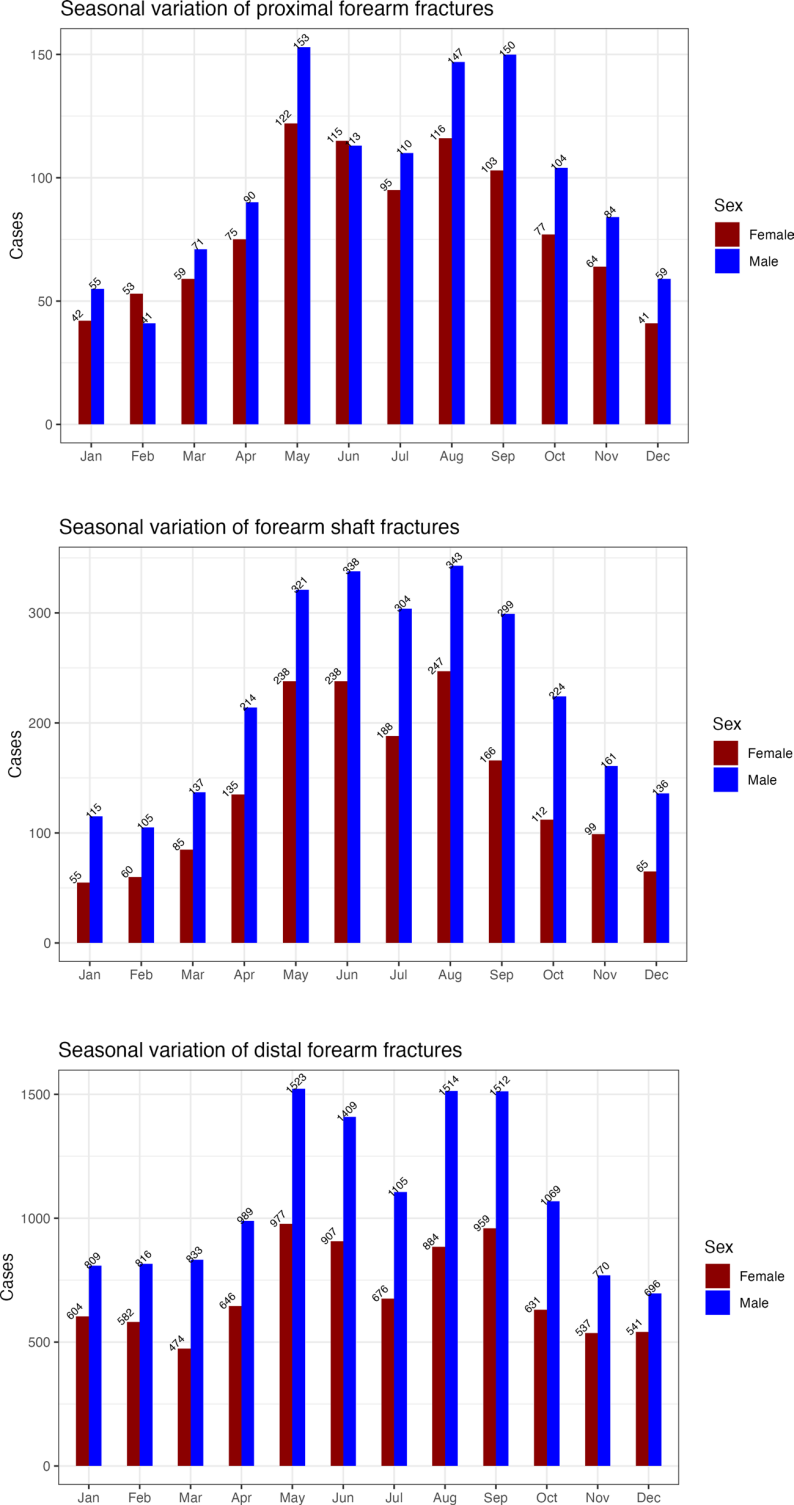
The seasonal variation of all pediatric forearm fractures registered in the SFR 2015-2019

31.6% of all fractures occurred in the summer, with a significant decrease in July. Spring accounted for 25.5% of the fractures, while autumn 25.4%, and winter 17.4% (Fig. 2). Summer was the leading season for forearm fractures across all ages and both sexes, except in the 12–16 age group, where spring had the highest incidence.

### Initial treatment

**Fig. 3:**
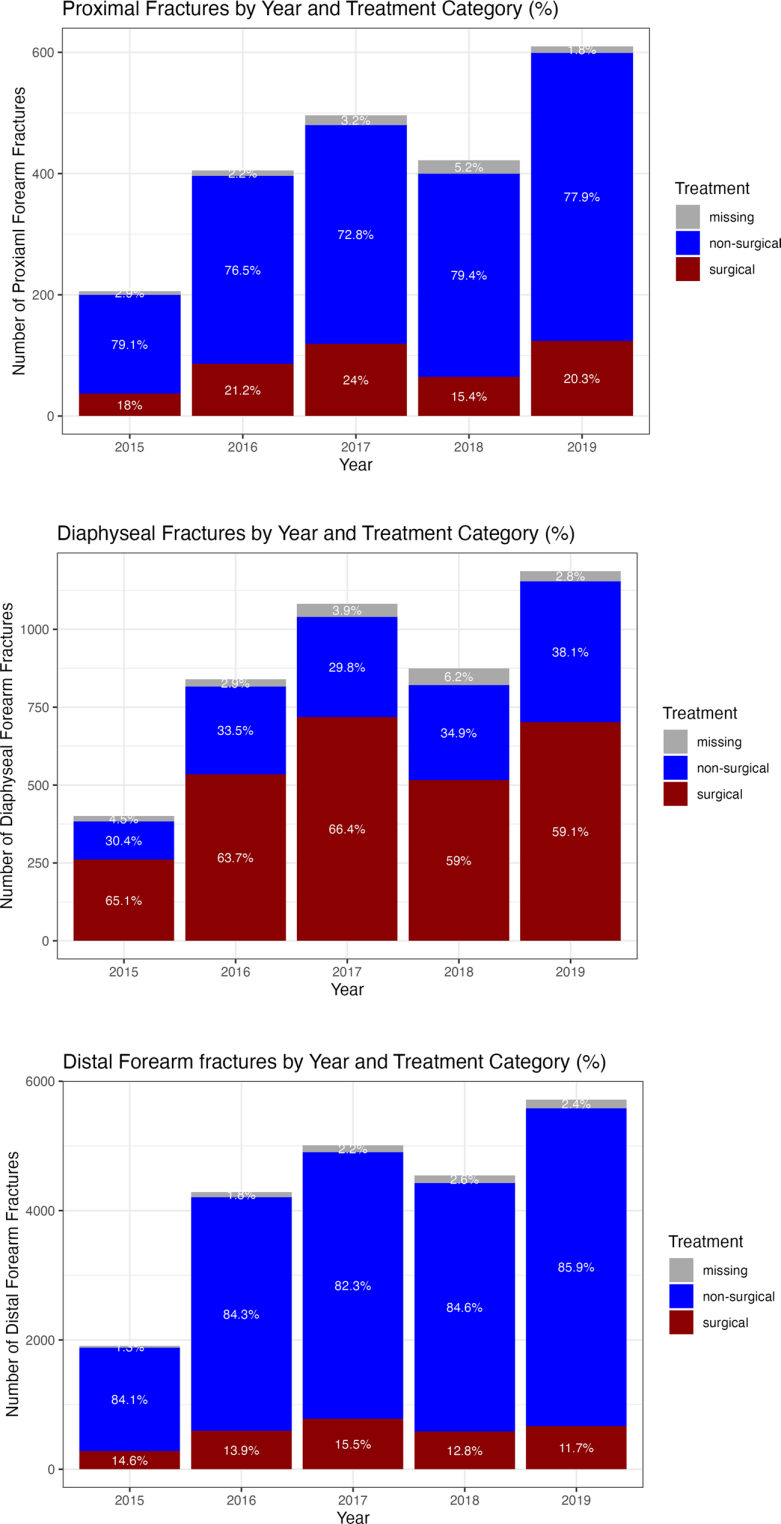
Distribution of Pediatric Forearm Fractures by Year and Treatment Type for each anatomical segment. SFR 2015-2019

About 76% of the fractures were treated non-surgically with or without reduction, with the highest rate of surgically treated fractures in the shaft segment (62%). There was no significant change of the treatment methods during the study period (Fig. 3) despite in 2015 when the SFR has been introduced and not all departments were reporting.

The most common surgical method used was fixation with pins, with 32.6% of all surgical procedures. Table 5 demonstrates the distribution between non-surgical and surgical treatment used for each fractured segment, including the specific surgical method used. Fractures sustained by girls were less likely to need surgical treatment (Odds ratio (OR): 0.82, Cl: 0.75–0.87) compared to boys. Diaphyseal forearm fractures had the highest overall risk for surgery (OR: 6.78, 95% CI: 6.01–7.64), and distal forearm fractures had the lowest risk (OR: 0.6, 95% CI: 0.54–0.67) compared to proximal forearm fractures.

**Table 5:** Distribution of treatment and specific surgical methods for pediatric forearm fractures registered in the SFR 2015-2019

			Proximal forearm(*n* = 2 139)	Diaphyseal forearm(*n* = 4 385)		Distal forearm(*n* = 21 463)	Overall(*n* = 27 987)			
*Missing data*			*65 (3.0%)*	*171 (3.9%)*		*466 (2.2%)*	*701 (2.5%)*			
**Non-surgical**			1 644 (76.9%)	1 482 (33.8%)		18 095 (84.3%)	21 221 (75.8%)			
**Surgical treatment**			**Proximal forearm** **N= 431 (20.1%)**	**Diaphyseal forearm** **N= 2732 (62.3%)**	**Distal forearm** **N= 2902 (13.5%)**			**Overall** **N= 6065 (21.7%)**		
External fixation			0 (0%)	2 (0.1%)		7 (0.2%)	9 (0.1%)			
Pin fixation			179 (41.5%)	94 (3.4%)		1702 (58.6%)	1975 (32.6%)			
Intramedullary nail/ESIN			55 (12.8%)	1451 (53.1%)		47 (1.6%)	1553 (25.6%)			
Plate fixation			14 (3.2%)	77 (2.8%)		62 (2.1%)	153 (2.5%)			
Screw fixation			21 (4.9%)	0 (0%)		0 (0%)	21 (0.3%)			
Combined surgical methods*			66 (15.3%)	437 (16.0%)		17 (0.6%)	520 (8.6%)			
Other surgical methods**			13(3.1%)	5 (0.2%)		6 (0.2%)	24 (0.4%)			
Closed reduction of the other bone			37 (8.6%)	46 (1.7%)		0 (0%)	83 (1.4%)			
Non-surgical of the other bone			46 (10.7%)	620 (22.7%)		1061 (36.6%)	1727 (28.5%)			

Note: *Including open reduction,* operation of tendon/muscle/ligament/joint capsule*,* combo of plate/pins or pins/screws and unspecified combo*

** Including osteosynthesis and different treatment of both-bone fractures

Regarding the risk for surgery for each fractured segment, there were no statistically significant differences between the ages and sexes for proximal forearm fractures. The risk for surgery of diaphyseal forearm fractures rose slightly with age (OR: 1.06, 95% CI 1.04–1.08) and was higher for girls (OR: 1.24, 95% CI 1.09–1.4). For the distal forearm fractures, there was no significant difference between the ages, but between the sexes, where girls had a lower risk for surgery (OR: 0.69, 95% CI 0.63–0.74) compared to boys.

## Discussion

### Main findings

The distal forearm was the most frequently affected segment, with torus fractures being the most common fracture type. Fractures were more common in boys than girls, and boys were generally older at the time of injury. Falls were the leading cause of fractures, most commonly occurring at sports facilities. Summer emerged as the peak season for forearm fractures. The majority of the fractures were managed non-surgically.

## Age and sex

International studies have reported sex-specific peak ages for forearm fractures. In the United Kingdom (UK), Cooper et al. found peak ages of 12–13 years for boys and 10–11 years for girls [1], consistent with findings by Valerio et al. in Italy [21]. Our results align with these and previous Swedish studies [3, 5, 8, 22–24] showing peak ages of 12–13 years in boys and 10 years in girls.

Similarly to other studies, we found that forearm fractures predominantly occurred in boys [1, 3, 25]. However, Landin observed a boy-to-girl ratio of 2:1 for shaft fractures [22], compared to our somewhat lower ratio of 1.5:1. This discrepancy may reflect differences in period, setting, and population characteristics, as Landin’s study was conducted at a single hospital in Malmö from 1950 to 1979, while our data are based on a national registry from 2015 to 2019, possibly capturing shifts in behavior with more active girls nowadays and activity patterns over time. An even higher proportion of 70% boys, was found in a German and Chinese study on hospitalized pediatric upper limb fractures [4, 25]. Although our data did not include hospitalization status, diaphyseal fractures in our cohort more often required surgical treatment, which may explain the higher proportion of boys in both cohorts.

### Segment and injury type

As in previous studies, the distal forearm [3, 5, 26, 27] was the most common fracture segment in our cohort followed by diaphyseal and proximal forearm fractures. However, comparisons with other studies are limited, as many analyze all pediatric forearm fractures without distinguishing between the segments. Buckle/torus fractures in the distal radius are described to be the most common type of fracture in children [28], and this was in alignment with our findings. Our data also indicated that buckle/torus fractures were the most common injury type in the proximal segment. The engagement of the growth plate with classification according to Salter-Harris was somehow lesser frequent than in the study of Liebs et al. [29] who described an engagement of the physis in more than 15% only in the distal forearm.

### Injury mechanisms

Pediatric forearm fractures occur from a range of injury mechanisms, with falls consistently identified as the most common cause worldwide [3, 5, 9, 21, 27]. Rennie et al. [9] suggest that falls from heights lower than a bed (< 1 m) were a common injury mechanism for distal radius fractures. Other common injury mechanisms that caused forearm fractures were falls >1 m, traffic accidents, sports-related, and falls down stairs or slopes [9]. Our findings align with this, showing that falls were the leading cause across all forearm segments (distal, diaphyseal, and proximal). There are more and more programs for learning how to fall in the elderly population for fracture prevention [30, 31], however as our and other data suggests it might be important even in children. To our knowledge only one RCT-study involved children in an 8-week learning-to-fall program and found positive effects on injury prevention [32].

Trampoline-related injuries have been highlighted as a significant contributor to diaphyseal fractures [33, 34]. Sinikumpu et al. [34] reported that the most common recreational injury cause for diaphyseal forearm fractures was related to trampolines, causing 25% of all fractures [1]. While trampoline use was not recorded as a distinct category in the SFR during the study period, it is likely included under “play equipment”—a term not clearly defined, potentially leading to varied interpretation. Nevertheless, trampolines are a probable contributor to these injuries in Sweden. One can argue that recommendations for using safety nets and advising that only one child should jump at a time could reduce the risk for fractures.

We also found that sports-related fractures increased with age, which is in line with previous research [3, 9]. Organized sports typically begin between the ages of 5 and 7 [3]. Rennie et al. noted that sports accounted for 12.1% of all pediatric fractures, with 26.7% affecting the distal radius, commonly linked to football [9, 35]. In Sweden, nearly 60% of children aged 12–18 are active in sports clubs, with football, floorball, and ice hockey being the most popular [35]. Our results showed that sports were the second most common injury cause, underscoring the importance of injury prevention strategies in youth sports.

We found 14 patients with assault as injury mechanism. All patients in this category were older than 7 years old. According to Randall et al., the most common site for fracture caused by abuse were of the femur and humerus. The majority of the children in this category were younger than 2 years old [36].

### Seasonal variation

The occurrence of pediatric fractures seemed to vary throughout the year.

Research from Scandinavia on all pediatric fractures indicates that the peak fracture occurrence was during the summer and spring [7, 10]. Our data aligned with these findings, along with the notable decrease in fractures rates in July [7, 10, 11, 22]. Many authors have discussed the cause to this decrease. Segal et al. [12] suggest that four factors contribute to this: (1) Behavioral shift during summer vacation; (2) Temperatures above 28 °C prevents outdoor play; (3) Travels abroad during summer vacation; (4) Weather-related effects on bone health. Lund et al. [10] gives other suggestions and argue that the decrease in fracture occurrence in July may be due to that most organized sports are paused during the summer vacation. Since sports-related activities are a common cause to pediatric fractures, this may be the reason for the drop in fracture incidence in July. Similar to other Swedish studies [3, 11], December was the month with the lowest fracture occurrence.

### Initial treatment

The golden standard treatment of pediatric forearm fractures is closed reduction and/or casting [3]. Surgical treatment is indicated when alignment cannot be achieved with non-surgical methods. Our data suggest that the most common initial treatment for pediatric forearm fractures in Sweden is indeed non-surgical despite diaphyseal fractures, which are predominantly treated surgically. The trend toward surgical treatment of forearm fractures, as described by Hansen et al., [16] could not be observed during our study period. A possible explanation for this could be that our study is subsequent to the study of Hansen et al., and the levels might have already been considered as high, but are not further increasing. According to Sinikumpu et al. [37], the complication rate is higher after the non-surgical approach. The authors claim that the most common complication was fracture re-displacement and was the dominant indication for re-operation.

The treatment for distal forearm fractures is going through changes. Perry et al. [28] investigated the outcome of rigid immobilization and soft bandaging treatment of torus fractures and found no significant difference in either pain or recovery of function between the two treatments. The authors suggest that treatment with a bandage and immediate discharge is safe, as there were no cases of worsened deformity, and it is an easy treatment that could be used worldwide. However, they highlight the need for educational efforts between the patient’s family and the physicians, as the families often seem to favor rigid immobilization [4]. Similarly, Marson et al. [38] investigated non-surgical treatment in completely displaced distal radius fractures, finding no differences in the clinical outcome. The multicenter prospective randomized noninferiority trial by Metcalfe et al. [39] (CRAFFT-Trial) is still ongoing.

### Strengths and limitations

A great strength of this study is the large study population of 26,587 patients with nearly 28,000 fractures. The SFR is a nationwide register covering about 80% of Sweden’s orthopedic units at our time of study, covering small countryside hospitals and university hospitals.

The completeness of the SFR compared with the National Patient Register (NPR) for pediatric forearm fractures was about 50% in 2019, and had increased from the start due to the step-wise introduction of SFR, with varying completeness across different hospitals [40]. However, the NPR-data are less suitable for an epidemiological study since they lack detailed information on injury site, injury mechanism, and fracture classification, and sometimes include even suspected fractures which later (after radiographs) have been ruled out but still appear in the NPR. The SFR has added trampoline as an injury cause as of late 2024, which could be interesting to explore in future studies.

Another key strength is the minimal amount of missing data—only a small percentage of registered fractures lacked details on injury mechanism, classification, or treatment. Classifications were made by the treating orthopedic surgeon, and previous validation studies have shown these to be generally accurate [18, 41–43]. 

Furthermore, as with all register-based studies, reporting bias can be a problem and contribute to incomplete or missed data. Furthermore, the lack of radiographic images made it impossible to validate the fractures, but the validation of the acetabular fracture classification in the SFR in 2022 [42] showed moderate accuracy, whereas the expert group had substantial to perfect inter- and intrarater agreement. Another limitation of register-based studies is the potential for misinterpretation of variables during data entry. Some units only report pediatric fractures that are treated surgically, which can introduce selection bias and overestimate the rate of surgical treatment. While the SFR includes data on reoperations, this falls outside the scope of our epidemiological study. Additionally, reoperation data require validation through medical records to ensure accuracy.

In the SFR, assault by another person can be specified. However, child abuse is probably not the first cause of injury coming to mind when registering, one could argue that there is a hidden number of children with this kind of injury mechanism. The initial registered injury mechanism can be corrected in the SFR if the suspicion of abuse is verified. In addition, the injury mechanism “assault by another person” is classified together with injuries sustained in a fight. Therefore, there is no distinct category specific for abuse which can lead to misinterpretation.

## Conclusion

In conclusion, most pediatric forearm fractures occurred in the distal forearm. The fracture risk was higher in boys and older children, while girls were more likely to undergo surgical treatment. Older children were often injured during sports and had a higher risk of undergoing surgical treatment. Younger children often sustained fractures during play. Most fractures were treated non-surgically, except diaphyseal fractures, where 62% required surgery. The rate for surgical treatment was stable during the study period. Fracture rates peaked in summer, with a dip in July, possibly due to vacations and reduced organized sports. These findings underline the need for improved safety in sports and play, and for educational efforts to improve falling skills targeting both children and parents. Further research should explore the impact of such education and protective equipment on injury rates and the outcome of non-surgical treatment in the long term.

## Data Availability

The dataset used in this study is not publicly available due to confidentiality requirements. While data sharing is possible, it is restricted under Swedish law (Public Access and Secrecy Act, Chapter 21 §7 and Chapter 25 §1). Interested parties may contact Uppsala University or the corresponding author for potential access in accordance with these laws. Data requests may also be submitted to the Center of Registers, Västra Götaland (http://registercentrum.se/) with approval from the Swedish Ethical Review Authority.
